# Newly Isolated *Paenibacillus monticola* sp. nov., a Novel Plant Growth-Promoting Rhizobacteria Strain From High-Altitude Spruce Forests in the Qilian Mountains, China

**DOI:** 10.3389/fmicb.2022.833313

**Published:** 2022-02-18

**Authors:** Hui-Ping Li, Ya-Nan Gan, Li-Jun Yue, Qing-Qing Han, Jia Chen, Qiong-Mei Liu, Qi Zhao, Jin-Lin Zhang

**Affiliations:** ^1^Key Laboratory of Grassland Livestock Industry Innovation, Ministry of Agriculture and Rural Affairs, College of Pastoral Agriculture Science and Technology, Lanzhou University, Lanzhou, China; ^2^Center for Grassland Microbiome, State Key Laboratory of Grassland Agro-Ecosystems, Lanzhou University, Lanzhou, China

**Keywords:** *Paenibacillus*, PGPR, novel species, qilian mountains, spruce

## Abstract

Species in the genus *Paenibacillus* from special habitats have attracted great attention due to their plant growth-promoting traits. A novel plant growth-promoting rhizobacteria (PGPR) species in the genus *Paenibacillus* was isolated from spruce forest at the height of 3,150 m in the Qilian Mountains, Gansu province, China. The phylogenetic analysis based on 16S rRNA, *rpoB*, and *nifH* gene sequences demonstrated that strain LC-T2*^T^* was affiliated in the genus *Paenibacillus* and exhibited the highest sequence similarity with *Paenibacillus donghaensis* KCTC 13049*^T^* (97.4%). Average nucleotide identity (ANIb and ANIm) and digital DNA–DNA hybridization (dDDH) between strain LC-T2*^T^* and *P. donghaensis* KCTC 13049*^T^* were 72.6, 83.3, and 21.2%, respectively, indicating their genetic differences at the species level. These differences were further verified by polar lipids profiles, major fatty acid contents, and several distinct physiological characteristics. Meanwhile, the draft genome analysis provided insight into the genetic features to support its plant-associated lifestyle and habitat adaptation. Subsequently, the effects of volatile organic compound (VOC) emitted from strain LC-T2*^T^* on the growth of *Arabidopsis* were evaluated. Application of strain LC-T2*^T^* significantly improved root surface area, root projection area, and root fork numbers by 158.3, 158.3, and 241.2%, respectively, compared to control. Also, the effects of LC-T2*^T^* on the growth of white clover (*Trifolium repens* L.) were further assessed by pot experiment. Application of LC-T2*^T^* also significantly improved the growth of white clover with root fresh weight increased over three-folds compared to control. Furthermore, the viable bacterial genera of rhizosphere soil were detected in each treatment. The number of genera from LC-T2*^T^*-inoculated rhizosphere soil was 1.7-fold higher than that of control, and some isolates were similar to strain LC-T2*^T^*, indicating that LC-T2*^T^* inoculation was effective in the rhizosphere soil of white clover. Overall, strain LC-T2*^T^* should be attributed to a novel PGPR species within the genus *Paenibacillus* based on phylogenetic relatedness, genotypic features, and phenotypic and inoculation experiment, for which the name *Paenibacillus monticola* sp. nov. is proposed.

## Introduction

Plant growth-promoting rhizobacteria (PGPR) enhance growth and health of host plants through various mechanisms, including phosphate solubilization, nitrogen fixation, siderophore production, synthesis of phytohormone, and emission of volatile organic compounds (VOCs) (Ryu et al., 2003; Fürnkranz et al., 2012; Grady et al., 2016; He et al., 2021). Many PGPRs also prevent rhizosphere colonization of pathogenic or parasitic organisms by secreting antagonistic compounds and inducing plant defenses and/or competition for nutrients (Backer et al., 2018; Woo and Pepe, 2018; Naamala and Smith, 2021). These valuable characteristics can help to reduce the dependence of agricultural production on chemical fertilizers and insecticides, maximize the ecological benefits, and accelerate the emergence of their applications in biotechnological processes (Backer et al., 2018; Woo and Pepe, 2018; Rani et al., 2021). Globally, species of the genus *Paenibacillus* rank the top among PGPRs in agriculture and horticulture (Kiran et al., 2017; Daud et al., 2019; Liu et al., 2021). Many studies showed that some species in the genus *Paenibacillus* can promote the growth of host plants (Fürnkranz et al., 2012; Ker et al., 2012; Grady et al., 2016; Kumari and Thakur, 2018; Liu et al., 2021). In addition, inoculation of plants with *Paenibacillus* sp. strains also produce novel bioactive metabolites for biological control and industrial applications (Daud et al., 2019). Khan et al. (2007) and Aw et al. (2016) highlighted that *Paenibacillus* species effectively improved the growth of tomato (*Lycopersicon esculentum*) and had antibacterial activity against a wide spectrum of pathogens. Therefore, species of the genus *Paenibacillus* has enormous potential as PGPR. However, only a few species of *Paenibacillus* have been explored in detail concerning their effects on the growth of forage crops.

White clover (*Trifolium repens* L.) is a considerable legume forage crop with strong adaptability, wide distribution, and easy cultivation (Ballhorn and Elias, 2014). It is suitable for silaging, haying, and grazing for livestock due to its high quality (Acharya et al., 2011). Meanwhile, white clover can also maintain soil fertility by providing nitrogen from its symbiotic interactions with rhizobia (Shamseldin et al., 2021). Additionally, it has well-developed and numerous stolons and/or shoots that are beneficial for water and soil conservation (Ballhorn and Elias, 2014; Zhang et al., 2020). A previous study showed that inoculation with *Bacillus amyloliquefaciens* GB03 significantly increased plant growth and biomass of white clover under both non-saline and saline conditions (Han et al., 2014). However, the effects of PGPR strains of *Paenibacillus* on the growth of white clover are unknown.

The Qilian Mountains are hydrologically and ecologically vital unit, as it functions as the water source for the irrigation agriculture in the Hexi Corridor and also maintains the ecological viability in the northern Alxa Highland (Zhao et al., 2009). The Qilian Mountains cover a large area with a complex topography, changeable climate types, and large numbers of plant species with obvious differences in spatial distribution (Liu et al., 2004; Zhao et al., 2009). The altitude ranges from 1,173 to 5,546 m (Wu and Jiang, 1998; Liu et al., 2004). Spruce is the dominant tree species and generally distributed at 2,400–3,400 m (Zhao et al., 2009). The Qilian Mountains are one of the most challenging habitats and abundant ecosystems and have been attracting tremendous attention in the fields of agriculture, ecology, and biotechnology. Recently, extensive research focused on soil nutrient characteristics, community structure, and microbial diversity in the Qilian Mountains (Zhao et al., 2015; Zhu et al., 2017; Jian et al., 2020; Lan et al., 2020). The bacterial genus *Paenibacillus* was detected in the microbiomes of different moss species in the Qilian Mountains with plant-promoting traits (Lan et al., 2020), indicating that strains of the genus *Paenibacillus* with plant growth-promoting traits existed in the Qilian Mountains.

The genus *Paenibacillus* was classified by Ash et al. (1991, 1993) by distinguishing members of the “16S rRNA group 3” bacilli from other lineages in the genus *Bacillus*. Subsequently, taxonomic characteristics of the genus *Paenibacillus* were further revised by Shida et al. (1997) and Padda et al. (2017). Until now, 337 species have been identified in the genus *Paenibacillus*, and the type species of this genus is *Paenibacillus polymyxa*.^1^ The DNA G + C content of species in *Paenibacillus* ranges from 39 to 54 mol% (Kiran et al., 2017). Anteiso-C_15:0_ is the predominant cellular fatty acid, and menaquinone-7 (MK-7) is the major respiratory quinone (Ashraf et al., 2017; Padda et al., 2017). Several species of the genus were also found to produce chitinases (Fu et al., 2014; Loni et al., 2014). Members of the genus have been isolated from various habitats, including desert (Lim et al., 2006a,b), agricultural soil (Kim et al., 2015), rhizosphere (Son et al., 2014), honeybee larvae (Genersch et al., 2006), human feces (Clermont et al., 2015), milk (Scheldeman et al., 2004), fresh water (Baik et al., 2001; Bae et al., 2010), warm springs (Chou et al., 2007), and eutrophic lake and glacier (Montes et al., 2004; Kishore et al., 2010), etc.

Thus, this study was aimed to explore PGPR resources of the genus *Paenibacillus* from spruce forests in Qilian Mountains. A bacterium strain, designated as LC-T2*^T^*, was isolated from high-altitude spruce forests in the Qilian Mountains. The taxonomic status of strain LC-T2*^T^* was evaluated based on phenotypic, phylogenetic, genotypic, and chemotaxonomic data. Furthermore, the plant growth-promotion effects of strain LC-T2*^T^* was assessed in *Arabidopsis* and white clover (*T. repens* L.). Our work indicated that novel species in *Paenibacillus* isolated from spruce forests in Qilian Mountains have potential application values in cultivation of legume crops.

## Materials and Methods

### Sample Collection and Microorganisms Isolation

Soil samples were collected from forests of the Qilian Mountains, Gansu province, China (38°25′32″N, 99°55′40″E, 3,150 m). Spruce was the dominant tree species at the altitude of 3,150 m in the Qilian Mountains. The soil sample was serially diluted with sterile 0.9% NaCl (*w*/*v*) solution, and dilutions were spread on tryptone soya agar (TSA). All plates were incubated aerobically at 25°C for 7 days. Morphologically different single colonies were randomly picked and further purified. Finally, the purified isolates were preserved as a glycerol suspension (20%, *v*/*v*) at −80°C.

### Phylogenetic Analysis

The genomic DNA of the isolate was extracted by Bacterial Genomic DNA Extraction kit (TianGen Biotech Co., Ltd., Beijing, China) according to manufacturer’s instructions. The 16S rRNA gene was amplified by PCR using a pair of universal primers, 27F and 1492R, as previous described (Li et al., 2020). The RNA polymerase β-subunit (*rpoB*) gene, an iconic housekeeping gene of the genus *Paenibacillus*, was amplified with primers *rpoB* 1698F (5′-AACATCGGTTTGATCAAC-3′) and *rpoB* 2041R (5′-CGTTGCATGTTGGTACCCAT-3′) (Yang Y.J. et al., 2018). The nitrogenase reductase (*nif*H) gene was amplified using the primers POLF (5′-TGCGAYCCSARRGCBGGYATCGG-3′) and POLR (5′-ATSGCCATCATYTCRCCGGA-3′) (Menéndez et al., 2017). The PCR product was purified by PCR purification kit (Sangon Biotech Co., Ltd., Shanghai, China) according to the manufacturer’s instructions. Cloning of the 16S rRNA gene was executed using a pMD 19-T Vector Cloning kit (Takara Bio., Inc., Otsu, Japan). Sequencing was performed by the Sanger method (Beijing AUGCT DNA-SYN Biotechnology Co., Ltd, Beijing, China). Then, the almost-complete 16S rRNA, *rpoB*, and *nifH* gene sequences were compiled with the program DNAMAN (version 8.0; Lynnon Biosoft, San Ramon, CA, United States) (Saitou and Nei, 1987). The EzTaxon-e server^2^ (Yoon et al., 2017) was used to calculate the levels of sequence similarity between strain LC-T2*^T^* and related type strains available in GenBank^3^ (Sayers et al., 2020). The phylogeny of 16S rRNA, *rpoB*, and *nifH* sequences was reconstructed by the neighbor-joining (NJ) (Saitou and Nei, 1987), maximum-likelihood (ML) (Felsenstein, 1981), and maximum-parsimony (MP) methods (Fitch, 1971) with MEGA 7.0 program (Kumar et al., 2016). Evolutionary distances were calculated using the Kimura’s two-parameter model, and bootstrap analysis was used to evaluate the tree topology by performing 1,000 replications (Felsenstein, 1985).

### Draft Genome Sequencing, Assembly, and Annotation

The draft genome shotgun project was sequenced using paired-end sequencing technology with the Illumina NovoSeq-PE150 platform (Novogene Biotech Co., Ltd., Tianjin, China). High-quality genomic DNA was carried out using Bacterial Genomic DNA Extraction kit (TianGen Biotech Co., Ltd., Beijing, China) according to standard protocol. The sequencing generated 1-Gb clean data. A *de novo* assembly of the reads was carried out using SOAPdenovo (version 2.04). The completeness of microbial genomes was assessed using the bioinformatics tool CheckM (Parks et al., 2015). The complete 16S rRNA gene sequence of strain LC-T2*^T^* was annotated *via* the RNAmmer 1.2 server (Lagesen et al., 2007) from the genome. The draft genome was annotated using the NCBI Prokaryotic Genome Annotation Pipeline (PGAP) (Tatusova et al., 2016; Haft et al., 2018). The predicted coding sequences (CDSs) and functional annotation were generated from the National Center for Biotechnology Information (NCBI) non-redundant database, Kyoto Encyclopedia of Genes and Genomes (KEGG), Cluster of Orthologous Groups of proteins (COG), and Gene Ontology (GO) databases. DNA G + C content was calculated from the draft genome sequence. BLAST algorithm (ANIb) and the MUMmer ultra-rapid aligning tool (ANIm) were used to calculate average nucleotide identity (ANI) by the JSpecies software tool available at the webpage.^4^ The digital DNA–DNA hybridization (dDDH) between strain LC-T2*^T^* and related reference strains was calculated by Genome-to-Genome Distance Calculator 2.1 (GGDC).^5^

### Morphological, Physiological, and Biochemical Taxonomic Analysis

The morphological, physiological, and biochemical characterizations such as growth in different bacteriological media, temperature, pH and NaCl concentrations, the Gram reaction, motility, oxidase, catalase, hydrolysis of Tween 80, DNA, casein, starch, and cellulase were carried out according to Li et al. (2020). Biochemical features were performed using the API 20NE, API ZYM, and API 50CH systems (bioMérieux). GENIII MicroPlates (Biolog) were used to check the utilization of 71 carbon sources as described by the manufacturer’s instructions. *Paenibacillus donghaensis* KCTC 13049*^T^*, a Xylan-degrading bacterial strain isolated from east sea sediment, and *Paenibacillus odorifer* JCM 21743*^T^*, a nitrogen-fixing strain isolated from wheat roots, were used as reference strains for comparative taxonomic characteristics (Berge et al., 2002; Choi et al., 2008). The two reference strains were obtained from the Korean Collection for Type Cultures (KCTC) and the Japan Collection of Microorganisms (JCM), respectively. Cells of strain LC-T2*^T^* and the reference strains cultured on Reasoner’s 2A (R2A) agar at 28°C were used for biochemical feature tests. For measurement of nitrogenase activity, strain LC-T2*^T^* and reference strains were grown on nitrogen-free medium (Zhuang et al., 2017). After 48 h at 28°C, strains were incubated in culture bottles with 10% (*v*/*v*) acetylene in air for 2 h and then analyzed for ethylene production by 450-GC gas chromatography (Berge et al., 2002).

For chemotaxonomic analysis, cells of strain LC-T2*^T^* and reference strains were routinely cultivated on R2A agar at 28°C and harvested at the mid-exponential growth phase. The fatty acid profiles were analyzed and identified by using the Microbial Identification System (Sherlock version 6.1; midi database, TSBA6) after saponification, methylation, and extraction, according to standard procedures (Sasser, 1990). The polar lipids were extracted and separated by a chloroform/methanol system and one- and two-dimensional thin-layer chromatography (TLC) as described previously (Minnikin et al., 1984; Kates, 1986). Total lipids were detected using molybdatophosphoric acid, aminolipids were detected using ninhydrin reagent, phospholipids were detected using molybdenum blue reagent, and glycolipids were detected using naphthol/sulfuric acid reagent (Minnikin et al., 1984; Kates, 1986). Respiratory quinones were extracted and purified from lyophilized cells, then analyzed by high performance liquid chromatography (HPLC) according to Collins’ method (Collins, 1985).

### Evaluation of Plant Growth-Promoting Abilities

Plate and pot experiments were used to evaluate plant growth-promoting capabilities of strain LC-T2*^T^*. Double-sterile distilled water (DDW) served as control. *Escherichia coli* strain DH5α and commercial *B. amyloliquefaciens* strain GB03 served as positive control. *Arabidopsis* seeds were surface sterilized with 70% ethanol for 3 min, washed with DDW several times, followed by 1% sodium hypochlorite for 10 min, finally thoroughly washed with DDW for 8–10 times, and then planted on one side of specialized plastic Petri dishes (100 × 15 mm) that contained a center partition; both sides contain half-strength Murashige and Skoog (MS) solid medium with 0.8% (*w*/*v*) agar and 1.0% (*w*/*v*) sucrose. Seeds were vernalized for 2 days at 4°C in the absence of light. Bacterial suspensions were prepared according to previously described methods (He et al., 2018). Cells were harvested from R2A plates, put into DDW to yield 1.0 × 10^9^ colony forming units (CFU) ml^–1^ as determined by optical density, and serially diluted with plate counts. Then, 10 μl of bacterial suspension was spotted at one side of the Petri dish and 2-day-old *Arabidopsis* seedlings were planted on the other side of the Petri dish. Seedlings were grown under growth chamber (Panasonic, Japan) with a 16/8-h light/dark cycle under 200 μmol m^–2^ s^–1^ total light intensity, a temperature of 22 ± 2°C, and a relative humidity of 50–55%. The root system was scanned by an EPSON scanner, and morphological parameters were analyzed using the root analysis system WinRHIZO (v5.0, Regent Instruments, Quebec, QC, Canada) after 14 days. In pot experiments, white clover (*T. repens* L.) seeds (presented by Wanhai Zhou at Gansu Agricultural University, China) were surface sterilized for 1 min in 70% ethanol followed by 10 min in 2% sodium hypochlorite; then, seeds were rinsed with sterile water for 10 times and germinated in filter paper for 3 days. The seedlings with uniform growth were transferred to a plastic pot (diameter 9 cm, depth 10 cm) containing autoclave-sterilized commercial vermiculite–soil mixture and watered with modified half-strength Hoagland’s solution three times per week. White clover seedlings were inoculated with 2 ml of prepared bacterial suspension culture as bacterial treatments or the same volume of DDW as control. Thirty-day-old plants were harvested for plant growth and physiological index measurements. When sampling, the rhizosphere soil samples were collected from the surface of root, and the culturable bacteria in rhizosphere were isolated again by multiple-dilution method to verify the effective inoculation.

### Data Analysis

Results of the growth and physiological parameters were showed as means with standard errors (*n* = 6). Statistical analysis was assessed by one-way analysis of variance (ANOVA) using SPSS statistical software (Ver. 19.0, SPSS Inc., Chicago, IL, United States). Duncan’s multiple range test was executed to detect a difference between means at a significance level of *P* < 0.05.

## Results

### Phylogenetic Analysis

**FIGURE 1:**
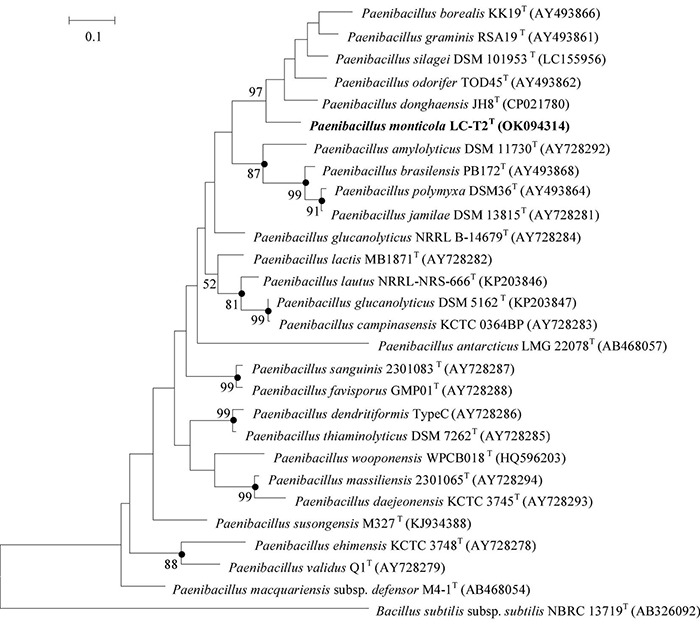
Maximum Likelihood phylogenetic trees based on partial *rpoB* gene sequences showing the relationships between strain LC-T2*^T^* and closely related species. *Bacillus subtilis subsp. subtilis* NBRC 13719*^T^* was used as the outgroups. Numbers at branching points are bootstrap values > 50%. Bar, 0.1 substitutions per nucleotide position. Filled circles indicate that the corresponding nodes were also formed in neighbor-joining and maximum-parsimony trees. *rpoB* gene sequences of *Paenibacillus donghaensis* JH8*^T^* was obtained from the genome sequence of strain *P. donghaensis* JH8*^T^*.

The complete 16S rRNA gene sequence (1,546 bp) was obtained from draft genome (GenBank accession number: OK058271). Comparative analysis built on 16S rRNA gene sequence revealed that strain LC-T2*^T^* was phylogenetically affiliated to the genus *Paenibacillus* in the family *Paenibacillaceae*. On the basis of phylogenetic analysis, the highest level of similarity was found between strain LC-T2*^T^* and *P. donghaensis* KCTC 13049*^T^* (97.4%), followed by *P. odorifer* JCM 21743*^T^* (96.8%) and other recognized members of the genus *Paenibacillus* (<96.7%). In the neighbor-joining phylogenetic tree, strain LC-T2*^T^* fell within the cluster comprising the *Paenibacillus* species and formed a distinct genetic lineage with *P. donghaensis* KCTC 13049*^T^* (Supplementary Figure 1) and likewise in the tree based on the ML and MP methods (data not shown). The *rpoB* gene fragment of strain LC-T2*^T^* (GenBank accession number: OK094314) shared 87.2% sequence identity with *P. donghaensis* KCTC 13049*^T^* and less than 84.8% identity with other members of the genus *Paenibacillus* (Figure 1). These data further confirmed that target lineage was belonging to the genus *Paenibacillus* and closely clustered with *P. donghaensis* KCTC 13049*^T^*. The comparison of the *nifH* gene sequence of strain LC-T2*^T^* (GenBank accession number: OK094315) with those of the type strains also showed that *P. donghaensis* KCTC 13049*^T^* was the most closely related living species, with a similarity value of 81.4%, followed by *P. odorifer* JCM 21743*^T^* (80.1%). The remaining available *nifH* sequences of the type species of the genus *Paenibacillus* showed less than 80% similarity to strain LC-T2*^T^*. The phylogenetic analysis of *nifH* indicated that strain LC-T2*^T^* clustered with *P. donghaensis* KCTC 13049*^T^* and was phylogenetically divergent from the cluster of any recognized species of the genus *Paenibacillus* (Supplementary Figure 2).

### Genome Characteristics of *Paenibacillus monticona* sp. nov. LC-T2*^T^*

Draft genome sequencing of strain LC-T2*^T^* (accession number: WJXB00000000) yielded a length of 7,082,651 bp with 35 contigs (total number > 500 bp) and N50 value of 657,675 after assembly. All contigs were larger than 536 bp, and the largest was 1,092,698 bp. The sequencing coverage was about ×200. A total of 6,236 genes were predicted, out of which 5,978 were protein-coding genes, 112 genes for RNA, and 146 pseudo genes (Supplementary Table 1). The DNA G + C content of strain LC-T2*^T^* was 46.0 mol%, which fell within the range given for species of the genus *Paenibacillus* (Yang D. et al., 2018). The pairwise ANIb, ANIm, and dDDH values between the genome of LC-T2*^T^* and four genomes of related species were 72.6–77.2, 83.3–84.3, and 20.4–23.0%, respectively. These values were obviously lower than the critical value of genomic species identification. The detailed results are displayed in Table 1.

**TABLE 1:** Average nucleotide identity (ANIb and ANIm) and DNA–DNA hybridization (DDH) values (%) of strain LC-T2*^T^* with phylogenetically related species of the genus *Paenibacillus*.

Species name	ANIb	ANIm	DDH
*Paenibacillus donghaensis* KCTC 13049^T^	72.6	83.3	21.2
*Paenibacillus odorifer* JCM 21743^T^	77.2	84.3	23.0
*Paenibacillus wynnii* DSM 18334^T^	75.1	83.7	20.4
*Paenibacillus borealis* DSM 13188^T^	74.3	83.7	21.0

### Insights From the Genome Sequence

Strain LC-T2*^T^* was isolated from high-altitude spruce forests in the Qilian Mountains. As shown in Table 2, the genome of strain LC-T2*^T^* contained a large number of genes that were related to plant growth and habitat adaptation, such as 10 genes coding for phosphate solubilization (*pyk*, *ppc*, *ackA*, *citA*, *citZ*, *aroK*, *ldh*, *phoP*, *phoR*, and *nudC*), four for auxin biosynthesis (*trpA*, *trpB*, *trpC*, and *trpS*), one for nitrogen fixation (*nifH*), and three for other processes of growth promotion (*speA*, *ilvH*, and *ilvB*), suggesting that strain LC-T2*^T^* had the ability to promote plant growth. The genome of strain LC-T2*^T^* contained several genes associated with secretion systems, biofilm formation, or motility. For instance, three genes responsible for flagellar motility (*motA*, *motB*, and *swrC*) and five genes responsible for chemotaxis (*cheA*, *cheY*, *cheR*, *cheB*, and *cheW*) indicated that strain LC-T2*^T^* could get attracted to or move toward nutrients and interact with plants. Additionally, extreme conditions, such as low temperature, hypoxia, alpine, strong ultraviolet, erosive forces, and thaw–freezing cycles, prevailed in the Qilian Mountains at high altitudes and shaped abundant extreme microorganisms. The genome of strain LC-T2*^T^* is well equipped with several genes that could alleviate the reactive oxygen species. Some genes responsible for superoxide dismutase [Mn] (*sodA*), superoxide dismutase [Fe] (*sodF*), and catalase (*katA*), demonstrated that strain LC-T2*^T^* could cope with rhizosphere oxidative environments. Notably, several genes of strain LC-T2*^T^* genome responded to extreme temperature at the height of 3,150 m in the Qilian Mountains. Genes coding for cold shock (*cspA*) and heat shock (*Hsp*20) showed that strain LC-T2*^T^* was able to adapt the temperature variation. In accordance with the data presented above, the draft genome analysis provided insights into the genetic features to support its plant-associated lifestyle and habitat adaptation.

**TABLE 2:** Putative gene identified in LC-T2*^T^* genome related to plant associated lifestyle and habitat adaptation.

Categories	Gene annotation	Gene numbers
Plant growth promotion	Phosphate solubilization	
	Pyruvate kinase (*pyk*)	2
	Phosphoenolpyruvate carboxylase (*ppc*)	1
	Acetate kinase (*ackA*)	1
	Citrate kinase (*citA/citZ*)	2
	Shikimate kinase (*aroK*)	1
	L-lactate dehydrogenase (*ldh*)	1
	Alkaline phosphatase (*phoP/phoR*)	6
	Nicotinamide adenine dinucleotide (NADH) pyrophosphatase (*nudC*)	1
	Auxin biosynthesis	
	Tryptophan synthase α chain (*trpA*)	1
	Tryptophan synthase β chain (*trpB*)	1
	Indole-3-glycerol phosphate synthase (*trpC*)	1
	Tryptophan–tRNA ligase (*trpS*)	1
	Nitrogen fixation	
	Nitrogenase iron protein (*nifH*)	1
	Others related to plant promotion	
	Arginine decarboxylase (*speA*)	1
	Acetolactate synthase small/large subunit (*ilvH*/*ilvB*)	2
Habitat adaptation	Plant rhizosphere environments	
	Flagellar motility (*motA*/*motB*/*swrC*)	3
	Chemotaxis (*cheA*/*cheY*/*cheR*/*cheB*/*cheW*)	12
	Oxidative stress alleviation	
	Superoxide dismutase [Mn] (*sodA*)	1
	Superoxide dismutase [Fe] (*sodF*)	1
	Catalase (*katA*)	1
	Cold and heat shock protein	
	Cold shock protein (*cspA*)	2
	Heat shock protein (*Hsp*20)	1
	Transcriptional regulator of stress and heat shock response (*ctsR*)	1

### Phenotypic and Biochemical Characteristics

**FIGURE 2:**
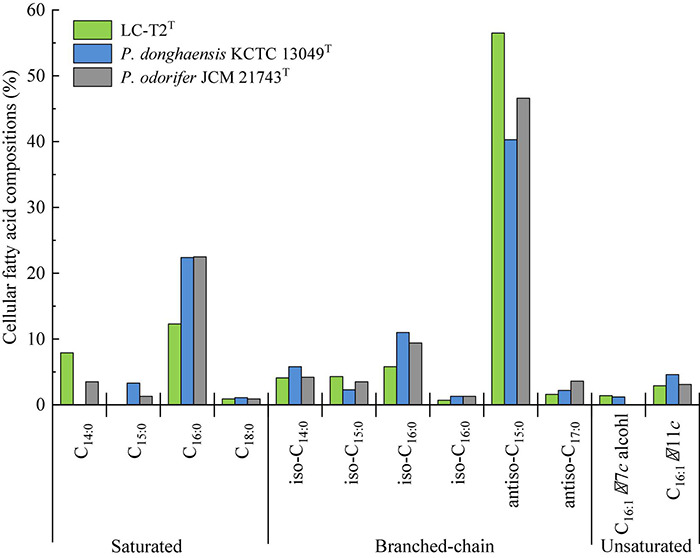
Cellular fatty acid compositions (%) of strain LC-T2*^T^* and the type strains of phylogenetically related species of the genus *Paenibacillus.*

The cell of strain LC-T2*^T^* was aerobic, Gram-negative (Supplementary Figure 3A), rod-shaped (4.2–4.5 × 0.6–0.7 μm) (Supplementary Figure 3B), and motile *via* peritrichous flagella (Supplementary Figure 3C). Colonies of strain LC-T2*^T^* on R2A agar were white, round, and smooth with approximately 0.5–1.5 mm in diameter after culture at 28°C for 3 days. It was able to grow aerobically at 4–32°C (optimum at 25–28°C), at pH 6.0–11.5 (optimum at 8.0–8.5), and with 0–1.5% (*w*/*v*) NaCl (optimum at 0%). Strain LC-T2*^T^* and the reference strains were positive for catalase and reduction of nitrate to nitrite, but they were negative for oxidase and hydrolysis of DNA, Tween 80, and cellulose. The detailed differential physiological and biochemical characteristics of strain LC-T2*^T^* and its closest type strains of the genus *Paenibacillus* are given in Table 3 and Figure 2. Strain LC-T2*^T^* was distinguished from the reference strains in API 20NE test strips: assimilation of glucose, mannitol, and *N*-Acetyl-glucosamine. Strain LC-T2*^T^* also differed from the closely related species in API ZYM test strips: cystine arylamidase, α-chymotrypsin, and acid phosphatase. Meanwhile, strain LC-T2*^T^* also distinguished from the reference-type species in API 50CH test strips: D-ribose and methyl-α-D-glucopyranoside, *N*-acetyl-glucosamine, and inulin test. In the aspect of nitrogenase activity, the amount of strain LC-T2*^T^* and reference strains, *P. donghaensis* KCTC 13049*^T^* and *P. odorifer* JCM 21743*^T^*, that could reduce acetylene to ethylene were 19.7, 15.5, and 25.4 (nmol C_2_H_4_) (mg protein)^–1^ h^–1^, respectively (Table 3).

**TABLE 3:** Characteristics that differentiate the novel species LC-T2*^T^* from phylogenetically related species of the genus *Paenibacillus*.

Characteristic	1	2	3
Habitat	Soil	Sediment	Rhizosphere
Temperature range (optimum) (°C)	4–32 (25–28)	4–30 (20–25)*	5–35 (30)^#^
pH range (optimum)	6–11.5 (8.0–8.5)	6–10 (ND)*	5.0–10.0 (ND)^$^
NaCl range (optimum)	0–1.5 (0%)	0–3.0 (ND)*	0–3.0 (ND)^$^
Assimilation of 20NE			
Glucose	−	w	+
Mannitol	+	−	−
*N*-Acetyl-glucosamine	–	–	+
Enzyme activity (API ZYM)			
Cystine arylamidase	−	w	−
α-chymotrypsin	−	−	w
Acid phosphatase	w	-	w
Acid production from API 50CH			
D-ribose	−	+	+
Methyl-α-D-glucopyranoside	+	−	+
D-mannose	+	w	w
*N*-acetyl-glucosamine	+	−	+
Inulin	−	−	+
Mannitol	+	+	−
D-melezitose	w	w	−
D-turanose	+	+	−
Nitrogenase activity [(nmol C_2_H_4_) (mg protein) ^–1^ h^–1^]	19.7 ± 1.6ab	15.5 ± 2.4b	25.4 ± 1.7a
DNA G + C content (mol%)	46.0	53.1*	44.0^#^

*Strains: 1, LC-T2^T^; 2, Paenibacillus donghaensis KCTC 13049^T^; 3, Paenibacillus odorifer JCM 21743^T^. Data for those strains are from this study, except as labelled. All strains were positive for motility, reduction of nitrate to nitrite, catalase, alkaline phosphatase, esterase (C4), esterase lipase (C8), leucine arylamidase, valine arylamidase, naphthol-AS-BI-phosphohydrolase, α-galactosidase, β-galactosidase, α-glucosidase, and β-glucosidase activities. Hydrolysis of aesculin, assimilation of: maltose. All strains were negative for lipase (C14), trypsin, β-glucuronidase, N-acetyl-β-glucosaminidase, α-mannosidase, and α-fucosidase activities and oxidase, hydrolysis of: DNA, Tween 80, and cellulose, assimilation of: mannose, arabinose, gluconate, citrate, adipic acid, capric acid, and phenylacetic acid. +, positive; −, negative; w, weakly positive; ND, no data available.*

*Data from Choi et al. (2008) and Jung et al. (2017).

*^#^Data from Berge et al. (2002).*

*^$^Data from Liu et al. (2018).*

The predominant cellular fatty acids (>10.0% of total fatty acids) of strain LC-T2*^T^* was identified as anteiso-C_15:0_ (56.5%) and C_16:0_ (12.3%) (Figure 2). The fatty acid profile of strain LC-T2*^T^* was similar to the reference strains, and all three species also contained anteiso-C_15:0_ (40.3–56.5%) and C_16:0_ (12.3–22.5%) as their major fatty acid. Moreover, the proportion of anteiso-C_15:0_ of strain LC-T2*^T^* was 2.4- and 1.2-fold higher than that of *P. donghaensis* KCTC 13049*^T^* and *P. odorifer* JCM 21743*^T^*, respectively, whereas the content of C_16:0_ of strain LC-T2*^T^* was nearly two-fold lower than that of the reference strains (Figure 2). The polar lipid pattern of strain LC-T2*^T^* was dominated by the presence of large amounts of diphosphatidylglycerol (DPG) and phosphatidylethanolamine (PE) and small amounts of phosphatidylglycerol (PG) and several unidentified ingredients as follows: three unidentified phospholipids (PL1–3), two unidentified aminophospholipids (APL1–2), and one unidentified glycolipid (GL1) (Supplementary Figure 4). MK-7 was detected as the only respiratory quinone in strain LC-T2*^T^*.

### Growth Promotion of *Arabidopsis thaliana* Exposed to Strain LC-T2*^T^*

**FIGURE 3:**
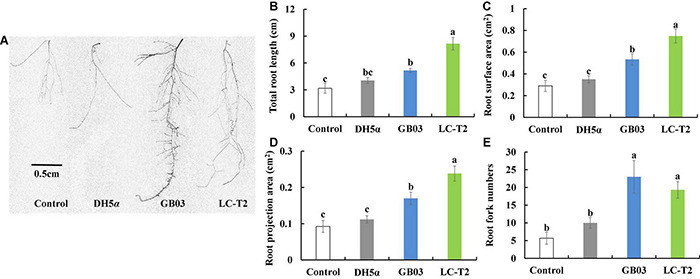
Effects of roots growth of *Arabidopsis* exposure to LC-T2*^T^* volatile organic compounds (VOCs). *Escherichia coli* (DH5α) and *Bacillus amyloliquefaciens* (GB03) as positive control l. **(A)** plant root image, **(B)** total root length, **(C)** root surface area, **(D)** root projection area, and **(E)** root fork numbers. Seedlings were taken image and root growth index were measured after 2 weeks exposure to *E. coli* (DH5α), *B. amyloliquefaciens* (GB03), and *Paenibacillus monticola* LC-T2*^T^*, respectively. Values are means and bars indicate SDs (*n* = 6). Columns with different letters indicate significant difference at *P* < 0.05 (Duncan test).

The apparent growth differences of *Arabidopsis* were observed between LC-T2*^T^* exposure and the other three treatments after 2 weeks of plant growth (Figure 3A). The significant differences were mainly observed on plant roots. Specifically, the total root length was significantly greater for LC-T2*^T^*-exposed roots (*P* < 0.05) by 61.0, 50.4, and 36.7% compared to control, DH5α, and GB03 exposure, respectively (Figure 3B). The highest root surface area and root projection area were also observed from exposure to LC-T2*^T^* VOCs. The root surface area was increased (*P* < 0.05) by 61.3, 53.1, and 28.8% (Figure 3C), and the root projection area was enhanced (*P* < 0.05) by 61.3, 53.1, and 28.7% (Figure 3D) compared to control, DH5α, and GB03 exposure, respectively. The root fork numbers was increased over 1.9-fold (*P* < 0.05) with LC-T2*^T^* VOCs compared to control and DH5α exposure, respectively. However, the root fork numbers of LC-T2*^T^* were little lower than GB03 exposure (Figure 3E).

### The Effect of Strain LC-T2*^T^* on the Growth of White Clover

**FIGURE 4:**
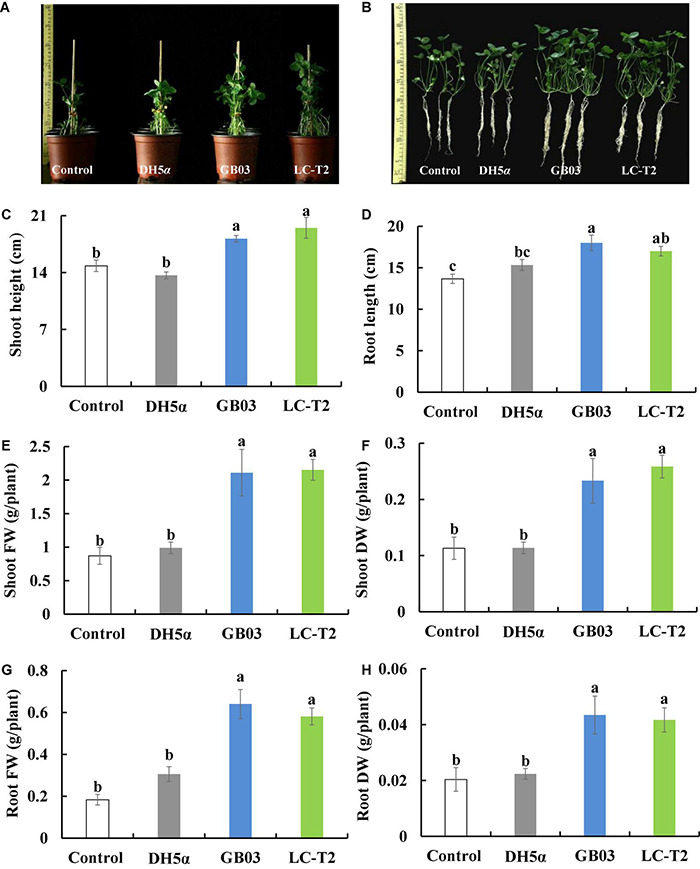
Effects of LC-T2*^T^* inoculation on seedling growth of white clover. *Escherichia coli* (DH5α) and *Bacillus amyloliquefaciens* (GB03) as positive control. **(A,B)** whole plant image, **(C)** shoot height, **(D)** root length, **(E)** shoot fresh weight, **(F)** shoot dry weight, **(G)** root fresh weight, and **(H)** root dry weight. Seedlings were taken image and biomass were measured after 30 days inoculate to bacterials suspension and double-sterile distilled water (DDW), respectively. Values are means and bars indicate SDs (*n* = 6). Columns with different letters indicate significant difference at *P* < 0.05 (Duncan test).

The influence of strain LC-T2*^T^* on the growth of white clover was further assessed. Shoot height was increased by 31.5 (*P* < 0.05), 42.7 (*P* < 0.05), and 7.3% compared to control, DH5α, and GB03 treatments, respectively (Figure 4C), and root length was increased by 24.4 (*P* < 0.05) and 10.9% compared to control and DH5α treatments, respectively (Figure 4D).

Plants inoculated with LC-T2*^T^* had a higher biomass than that of control, DH5α, and GB03. Shoot fresh weight was raised with the LC-T2*^T^* group by 147.5 (*P* < 0.05), 117.6 (*P* < 0.05), and 2.0% (Figure 4E) and shoot dry weight was increased with the LC-T2*^T^* group by 128.6 (*P* < 0.05), 127.1 (*P* < 0.05), and 10.8% compared to control, DH5α, and GB03 treatments, respectively (Figure 4F). Likewise, the root fresh weight of LC-T2*^T^*-inoculated plants was about 216.2 and 89.8% (Figure 4G) and the root dry weight of LC-T2*^T^*-inoculated plants was about 104.7 and 86.5% (Figure 4H) higher than those of the control and DH5α treatments, respectively (*P* < 0.05). However, the root fresh weight and root dry weight of LC-T2*^T^* were a little lower compared to GB03 exposure (Figures 4G,H).

Strain LC-T2*^T^* can also enhance the accumulation of plant biomass by root activity and chlorophyll content. Root activity was improved with the LC-T2*^T^* group by 57.7 and 60.2% compared to control and DH5α treatments, respectively (*P* < 0.05; Supplementary Figure 5A). The content of chlorophyll a of LC-T2*^T^*-inoculated plants was increased by 25.9 (*P* < 0.05), 27.1 (*P* < 0.05), and 1.8% compared to control, DH5α, and GB03 treatments, respectively (Supplementary Figure 5B), and the content of chlorophyll b was improved by 2.1- (*P* < 0.05), 1.2- (*P* < 0.05), and 1.0-fold with LC-T2*^T^* treatment compared to control, DH5α, and GB03 treatments, respectively (Supplementary Figure 5B).

In addition, the photosynthetic rate was enhanced with the LC-T2*^T^* group by 13.7 and 26.5% compared to control and DH5α treatments, respectively (Supplementary Figure 5C). The transpiration rate was raised by 1.2- and 1.5-fold (*P* < 0.05) with LC-T2*^T^*-inoculated plants (Supplementary Figure 5D), and the stomatal conductance was increased by 17.4 and 39.1% (Supplementary Figure 5E) compared to control and DH5α treatments, respectively. The photosynthetic rate, transpiration rate, and stomatal conductance of LC-T2*^T^*-inoculated plants were slightly lower than those of GB03 treatment, and differences were not statistically significant (Supplementary Figures 5C–E). The water-use efficiency was improved by 1.8- (*P* < 0.05), 1.2-, and 1.2-fold with LC-T2*^T^* treatment compared to control, DH5α, and GB03 treatments, respectively (Supplementary Figure 5F).

### Effectiveness of LC-T2*^T^* Inoculation

Culturable bacterial strains have been isolated from the rhizosphere after 20 days of inoculation to verify the existence of inoculated strains. The number of genera from LC-T2*^T^*-inoculated rhizosphere soil was 1.7-fold higher than that of control and DH5α, respectively. In the diversity and number of species, LC-T2*^T^*-inoculated soil was also superior to control and DH5α-inoculated soil (Table 4). Some isolates were similar to strain LC-T2*^T^* and GB03, respectively, except for DH5α treatment (Supplementary Tables 2, 3). The species in the genus *Bacillus* and *Pseudomonas* were also isolated from all inoculated soils, whereas a higher abundance of *Rhodococcus qingshengii* was found in control and DH5α-inoculated soils.

**TABLE 4:** The number of genera counted from isolates in the white clover rhizosphere soil.

Treatments	Control	DH5α	GB03	LC-T2
Number of genus	6	6	10	10
Genus	*Bacillus* *Flavobacterium Microbacterium Paenarthrobacter Rhodococcus* *Sphingobacterium*	*Microbacterium* *Paenarthrobacter* *Paracoccus* *Pseudomonas Rhodococcus* *Sphingomonas*	*Arthrobacter* Bacillus *Curvibacter* *Flavobacterium* *Leifsonia* *Paracoccus* *Paenarthrobacter* *Pseudomonas* *Pseudarthrobacter Rhodococcus*	*Arthrobacter* *Asticcacaulis* *Bacillus* *Exiguobacterium* *Flavobacterium Microbacterium* *Paenibacillus* *Pseudomonas* *Rhodococcus* *Sphingopyxis*

## Discussion

Researchers have always sought to isolate novel species of the genus *Paenibacillus* from different habitats. As an important ecological security barrier in western China, the Qilian Mountains possess abundant glacier, water, forest, grassland, and animal resources (Zhao et al., 2009). The rich and diverse natural eco-environments of the Qilian Mountains harbor unique and great diversity of microbial resources. In the current work, a novel *Paenibacillus* species from spruce forest at a high altitude of 3,150 m in the Qilian Mountains was isolated and characterized. A phylogenetic analysis of 16S rRNA gene sequence, one of the most powerful and frequently used methods for identification of bacteria (Busse et al., 1996), revealed that strain LC-T2*^T^* was a member of the genus *Paenibacillus* with the highest similarity to *P. donghaensis* KCTC 13049*^T^* (97.4%). To further define the phylogenetic affinity of strain LC-T2*^T^*, we also analyzed *rpoB* gene sequence, which was more discriminative than 16S rRNA gene sequence in distinguishing members of the genus *Paenibacillus* (da Mota et al., 2004). Nitrogen fixation-related genes are widely used as marker genes to analyze the phylogenetic relationship of nitrogen-fixing bacteria and archaea (Jacobson et al., 1989; Choo et al., 2003; Brigle et al., 2011; Gaby and Buckley, 2014). Most members of the genus *Paenibacillus* have been found to possess nitrogenase activity. Therefore, *nifH* gene sequence was also used to distinguish members of the genus *Paenibacillus*. In all phylogenetic trees (Figure 1 and Supplementary Figures 1, 2), strain LC-T2*^T^* was obviously different from all known taxa. In brief, the phylogenetic analysis based on 16S rRNA, *rpoB*, and *nifH* gene sequences highlighted that strain LC-T2*^T^* was assigned to a novel species in the genus *Paenibacillus*.

At the genomic level, this study provided more robust evidence to support the taxonomic status of strain LC-T2*^T^*. The dDDH value and ANI value between strain LC-T2*^T^* and its closest phylogenetic relatives were lower than 23.0 and 84.3%, respectively. Studies have shown that the cut-off of 70% genomic relatedness with dDDH was generally recommended for species delineation and has been found to correlate to 95–96% ANI (Richter and Rosselló-Móra, 2009; Meier-Kolthoff et al., 2013, 2014a,2014b). In the current work, both dDDH value and ANI value were significantly below the threshold for species circumscriptions. These data demonstrated that strain LC-T2*^T^* should be considered as the representative of a novel species of the genus *Paenibacillus.* Meanwhile, *rpoB* gene sequence similarity can provide efficient supplement to dDDH and ANI measurements to delineate bacterial species and genera, especially for *Paenibacillus* (Adékambi et al., 2008). On the basis of previous research, 97.7% sequence similarity of *rpoB* gene was, as a threshold for species delineation, correlated with a dDDH value < 70% and an ANI value < 94.3% (Adékambi et al., 2008). Here, we found that *rpoB* gene sequence identity of strain LC-T2*^T^* with the other *Paenibacillus* species was lower than 87.2%. In summary, the values of ANIb, ANIm, and dDDH between strain LC-T2*^T^* and the typical strains for the closest *Paenibacillus* species were obviously lower than the acceptable threshold for bacterial species definition (Table 1), indicating that strain LC-T2*^T^* represented a novel *Paenibacillus* species.

A classification of strain LC-T2*^T^* at species level was greatly supported by phenotypic, physiological, and chemotaxonomic data. As shown in Table 3, Figure 2, and Supplementary Figures 3, 4, the similarities and differences of strain LC-T2*^T^* and its closest typical strains in the genus *Paenibacillus* are presented. Members of *Paenibacillus* were known to be Gram-positive, variable, and negative (Aw et al., 2016). Strain LC-T2*^T^* was found Gram-negative during the whole culture period, which was significantly different from the two reference strains (Berge et al., 2002; Choi et al., 2008). The dominant fatty acids, polar lipid profiles, and respiratory quinone of strain LC-T2*^T^* were in accordance with those found in members of the genus *Paenibacillus* (Ash et al., 1993; Shida et al., 1997; Lim et al., 2006a,b; Priest, 2009; Huang et al., 2016; Ashraf et al., 2017; Yang D. et al., 2018; Yang Y.J. et al., 2018). However, differences were observed in proportion of these components, which enabled strain LC-T2*^T^* to be clearly distinguished. Additionally, the nitrogenase activity of strain LC-T2*^T^* was about 27.1% higher than that of *P. donghaensis* KCTC 13049*^T^* and 22.4% lower than that of *P. odorifer* JCM 21743*^T^* (Table 3). Previous studies suggested that *P. donghaensis* KCTC 13049*^T^* and *P. odorifer* JCM 21743*^T^* exhibited a weak nitrogenase activity (Berge et al., 2002; Jin et al., 2011; Xie et al., 2012; Zhuang et al., 2017). In the current work, nitrogenase activities of reference strains were in good agreement with the results of previous studies (Berge et al., 2002; Jin et al., 2011; Xie et al., 2012; Zhuang et al., 2017). Therefore, strain LC-T2*^T^* could also be classified as bacterial strains with a weak nitrogenase activity.

Previous models of rhizobacterial-stimulated plant growth promotion suggested that soil microbes can drive plant growth promotion *via* emission of volatile chemicals (Timmusk et al., 1999; Ryu et al., 2003; Zhang et al., 2007; Luo et al., 2020). In *Arabidopsis*, volatile emissions from GB03 can regulate auxin homeostasis, transport, and cell expansion (Ryu et al., 2003; Zhang et al., 2007). In this work, strain LC-T2*^T^* conferred an increased total root length, root surface area, root project area, and root fork numbers in Petri dish-grown Arabidopsis seedlings *via* emission of volatile chemicals (Figure 3). Interestingly, the root length of seedlings inoculated with LC-T2*^T^* was longer than that inoculated with GB03, but the number of root fork was less than that inoculated with GB03, which might be attributed to GB03 VOCs that could increase the root auxin content and auxin accumulation at the lateral root initiation sites (Zhang et al., 2007; Xie et al., 2009; Zamioudis et al., 2013). Studies showed that GB03 VOCs specifically regulated plant auxin homeostasis to accelerate leaf expansion. Conveniently, adventitious roots seemed to successfully offer such a balance (Zhang et al., 2007). However, in the present study, LC-T2*^T^* VOCs mainly promoted the plant growth through increasing root length rather than the number of adventitious roots in *Arabidopsis*. Combined with the data of LC-T2*^T^* genome, genes coding for the production of auxins were identified (Table 2). Therefore, it was inferred that a different mechanism existed in the regulation patterns of hormone-related genes between strain LC-T2*^T^* and GB03, which requires to be further verified. In addition, volatile metabolites from some species of the genus *Paenibacillus* could also activate induced systemic resistance (ISR) against pathogenic microorganisms (Timmusk et al., 2005; Kiran et al., 2017; Daud et al., 2019). However, whether the VOCs from strain LC-T2*^T^* could also prevent pathogenic microorganisms remains to be investigated.

The beneficial effects of PGPR arouse interests and have been studied in various plants over the past decades worldwide (Zhao et al., 2016; Brito et al., 2017; Backer et al., 2018; Kumari and Thakur, 2018). Various studies demonstrated that soil inoculation with PGPR can promote plant growth, increase crop yields, enhance plant stress tolerance, and augment reproductive success (Han et al., 2014; Niu et al., 2016; Ke et al., 2019). The interest in *Paenibacillus* has mounted up since many strains have been found to possess potential agronomic value (e.g., *Paenibacillus ehimensis*, *Paenibacillus alvei*, *P. polymyxa*, and *Paenibacillus riograndensis*) (Antonopoulos et al., 2008; Naing et al., 2014; Brito et al., 2017). Kumari and Thakur (2018) demonstrated that treatment with *Paenibacillus* sp. ISTP10 significantly improved root fresh weight (131%), shoot fresh weight (105.14%), and total chlorophyll content (77.85%) of cotton in Cd-contaminated soil. White clover (*T. repens* L.), as a kind of high-quality forage in northwest China, has been gradually recognized of its advantages (Zhang et al., 2020). Therefore, its yield and quality have attracted considerable attention. However, available studies involved in the interaction of white clover with *Paenibacillus* sp. were rarely reported. Here, the substantial effects of the novel species of *Paenibacillus* LC-T2*^T^* on the growth of white clover were further assessed after it was found to have a positive response on the roots of *A. thaliana*. The plant appearance became larger, and all physiological parameters showed rising tendency in varying degrees with LC-T2*^T^* treatment compared to the control and DH5α treatments (Figure 4 and Supplementary Figure 5). Noticeably, shoot weight and root weight of samples inoculated with LC-T2*^T^* were almost twice as higher compared to control and DH5α treatments (Figures 4E–H). Interestingly, shoot height and shoot weight of LC-T2*^T^*-inoculated plants were slightly higher than those of GB03-inoculated plants. However, root length and root weight of LC-T2*^T^* were slightly lower than GB03, which was similar to results from plate experiment. Kim et al. (2011) found that among the selected 20 representative PGPR, most of the recognized genera were *Paenibacillus*, *Bacillus*, and *Pseudomonas*, which could remarkably enhance plant height, stem diameter, and fresh weight of cucumber. However, there was no obvious correlation between different isolates on the growth of cucumber based on PGPR genetic diversity, which suggested that there were differences in the regulation mechanism of different strains on cucumber growth. Therefore, it was supposed that strain LC-T2*^T^* and GB03 had different regulation patterns on the shoot and root of plant growth promotion, which needs to be further explored.

The success of colonization in the rhizosphere was one of the prerequisites for microbial inoculants to exhibit their plant growth-promotion characteristics (Mosimann et al., 2017; Mawarda et al., 2020). In this work, numerous bacterial strains were found from the rhizosphere soil after 20-day inoculation, and the number of genera of the isolates from LC-T2*^T^*-inoculated soil was almost twice that of control and DH5α-inoculated soil (Table 4). Some isolates were found to be similar to strain LC-T2*^T^* (Supplementary Table 2). These results suggested that rhizosphere inoculation with LC-T2*^T^* was effective. Strain LC-T2*^T^* had the capability of rapidly adapting to the environment, recruiting more rhizobacteria, and inhibiting pathogenic bacteria, which were vital for host plant growth promotion (Schreiter et al., 2014; Ke et al., 2019). In addition, species belonging to the genus *Bacillus* and *Pseudomonas* were also found in all inoculated rhizosphere soils, indicating that the genus *Bacillus* and *Pseudomonas* were the dominant genera in the soil (Zaidi et al., 2009). Also, a kind of carbendazim-degrading bacterium species, *R. qingshengii*, were obtained from DH5α-inoculated rhizosphere soil that probably inhibited or inactivated other microorganisms while degrading carbendazim (Xu et al., 2007). These results demonstrated that strain DH5α had poor survival and colonization ability in the rhizosphere and was at a competitive disadvantage (Majidzadeh and Fatahi-Bafghi, 2018; Chen et al., 2019). In light of the above research results, we supposed that both LC-T2*^T^* VOCs and soil inoculation of LC-T2*^T^* could improve plant growth, and LC-T2*^T^* could be qualified as a kind of PGPR candidate for agricultural crop production.

## Conclusion

Sequence analysis of housekeeping genes (16S rRNA and *rpoB*) and *nif*H gene demonstrated that strain LC-T2*^T^* could be representative of a new species within the genus *Paenibacillus*. The dDDH, ANIb, and ANIm analyses confirmed this presumption with their values less than 23.0, 77.2, and 84.3%, respectively. The distinctness and potential beneficial functions of strain LC-T2*^T^* at the species level was also supported by genomic data. The taxonomic status of strain LC-T2*^T^* was further clarified according to the content of anteiso-C_15:0_ and C_16:0_ and the profiles of DPG, PE, and PG. The above results clearly located that strain LC-T2*^T^* is a novel species within *Paenibacillus*. This work also established that rhizosphere inoculation with strain LC-T2*^T^* could significantly increase plant growth of legume crops like white clover, which made strain LC-T2*^T^* a potential excellent PGPR strain for practical application in legume crops.

### Description of *Paenibacillus monticola* sp. nov.

*Paenibacillus monticola* (mon.ti’co.la. L. n. *mons*, -*ntis* mountain; L. suff. -*cola*, inhabitant; N.L. masc. n. *monticola*, living in the mountains).

Cells are Gram-stain-negative, rod-shaped (4.2–4.5 × 0.6–0.7 μm) and motile by means of peritrichous flagella. Colonies of strain LC-T2*^T^* were white, round, and smooth with approximately 0.5–1.5 mm in diameter after culture at 28°C for 3 days on R2A agar. The isolate grew well on R2A agar, ISP 2 agar, PYG agar, and TY agar and weakly on NA and TSA, but no growth occurs on MA, LB agar, and MacConkey agar. Growth of strain LC-T2*^T^* occurred at 4–32°C (optimum, 25–28°C), at pH 6.0–11.5 (optimum, 8.0–8.5), and with 0–1.5% (*w*/*v*) NaCl (optimum, 0%). Strain LC-T2*^T^* was positive for catalase, the reduction of nitrate to nitrite, and the assimilation of mannitol, but it was negative for oxidase and hydrolysis of cellulose, Tween 80, and DNA. In API ZYM test strips, strain LC-T2*^T^* was as follows: positive for alkaline phosphatase, acid phosphatase, esterase (C4), esterase lipase (C8), leucine arylamidase, valine arylamidase, naphthol-AS-BI-phosphohydrolase, α-galactosidase, β-galactosidase, α-glucosidase, and β-glucosidase activities and negative for lipase (C14), trypsin, β-glucuronidase, *N*-acetyl-β-glucosaminidase, α-mannosidase, cystine arylamidase, α-chymotrypsin, and α-fucosidase activities. Acid is produced from methyl-β-D-xylopyranoside, D-mannose, *N*-acetyl-glucosamine, mannitol, D-turanose, L-arabinose, D-cellobiose, D-lactose, D-raffinose, D-turanose, D-sucrose, arbutin, and esculin. The following compounds are utilized as sole carbon sources in the GENIII microplates: Dextrin, D-maltose, D-trehalose, D-cellobiose, gentiobiose, sucrose, D-turanose, stachyose, D-raffinose, α-D-lactose, D-melibiose, *N*-acetyl-D-glucosamine, α-D-glucose, D-mannose, D-fructose, D-galactose, D-sorbitol, and D-mannitol. The predominant cellular fatty acids (>10.0% of total fatty acids) of strain LC-T2*^T^* were anteiso-C_15:0_ and C_16:0_. The major polar lipids of strain LC-T2*^T^* were established as DPG, PE, PG, and several unidentified ingredient as follows: three unidentified phospholipids (PL1–3), two unidentified aminophospholipids (APL1–2), and one unidentified glycolipid (GL1). Menaquinone-7 (MK-7) was detected as the only respiratory quinone. The DNA G + C content is 46.0 mol%.

The type strain is LC-T2*^T^* (=CCTCC AB 2019254*^T^* = KCTC 43175*^T^*), isolated from spruce forest in the Qilian Mountains, Gansu province, China (38°25′32″N, 99°55′40″E). The GenBank/EMBL/DDBJ accession number for 16S rRNA, *rpoB*, and *nifH* gene sequence and the whole-genome sequence of strain LC-T2*^T^* can be found at: https://www.ncbi.nlm.nih.gov/genbank/, OK058271, OK094314, OK094315, and WJXB00000000, respectively.

## Data Availability Statement

The datasets presented in this study can be found in online repositories. The names of the repositories and accession numbers can be found below: https://www.ncbi.nlm.nih.gov/genbank/, OK058271 https://www.ncbi.nlm.nih.gov/genbank/, OK094314 https://www.ncbi.nlm.nih.gov/genbank/, OK094315 https://www.ncbi.nlm.nih.gov/genbank/, WJXB00000000.

## Author Contributions

H-PL and QZ designed the experiments. L-JY provided the soil samples. H-PL performed most work on isolating and identifying bacterial strains, being assisted by Y-NG, JC, and Q-ML and wrote the first draft of the manuscript. Y-NG and JC assisted in the completion of the plant inoculation experiment. Q-QH did the proofreading of the first version. QZ and J-LZ provided guidance in scientific knowledge and correction of grammatical errors. All authors contributed to the article and approved the submitted version.

## Conflict of Interest

The authors declare that the research was conducted in the absence of any commercial or financial relationships that could be construed as a potential conflict of interest.

## Publisher’s Note

All claims expressed in this article are solely those of the authors and do not necessarily represent those of their affiliated organizations, or those of the publisher, the editors and the reviewers. Any product that may be evaluated in this article, or claim that may be made by its manufacturer, is not guaranteed or endorsed by the publisher.

## Footnotes

1
http://www.bacterio.net/paenibacillus.html

2
http://www.ezbiocloud.net/apps

3
www.ncbi.nlm.nih.gov/genbank/

4
http://www.imedea.uib.es/jspecies

5
http://ggdc.dsmz.de/distcalc2.php
