# Supplementary material for: Newly Isolated Paenibacillus monticola sp. nov., a Novel Plant Growth-Promoting Rhizobacteria Strain From High-Altitude Spruce Forests in the Qilian Mountains, China
Source: Front Microbiol. 2022 Feb 18;13:833313. doi: 10.3389/fmicb.2022.833313 (PMC8895201; doi:10.3389/fmicb.2022.833313)
Supplement: Supplementary file 1 [file Data_Sheet_1.docx]

***Paenibacillus***

***Fontibacillus***

***Saccharibacillus***

***Chengkuizengellas***

***Marinicrinis***

***Gorillibacterium***

***Paludirhabdus***

***Xylanibacillus***

***Cohnella***

***Ammoniibacillus***

***Thermobacillus***

***Aneurinibacillus***

***Ammoniphilus***

***Oxalophagus***

***Brevibacillus***

**Supplementary Figure 1 |** Neighbor-joining tree showing the phylogenetic relationship of strain LC-T2^T^ among closely related members of the family *Paenibacillaceae*, based on 16S rRNA gene sequences. Bootstrap values (>70%) based on 1000 replications are shown at branch nodes. Filled circles indicate that the corresponding nodes were also formed in maximum-likelihood and maximum-parsimony trees, and 15 strains with type taxon of *Paenibacillaceae* are highlighted in blue. *Escherichia coli* ATCC 11775^T^ was used as an outgroup. Bar, 0.02 substitutions per nucleotide position.

**Supplementary Figure 2 |** Neighbor-joining phylogenetic trees based on partial *nifH* gene sequences showing the relationships between strain LC-T2^T^ and closely related species. *Mesorhizobium ciceri* UPM-Ca7^T^ was used as the outgroups. Filled circles indicate that the corresponding nodes were also formed in maximum-likelihood and maximum-parsimony trees. Numbers at branching points are bootstrap values >50 %. Bar, 0.1 substitutions per nucleotide position. *nifH* gene sequences of *Paenibacillus donghaensis* JH8^T^ was obtained from the genome sequence of strain *Paenibacillus donghaensis* JH8^T^.

**Supplementary Figure 3 | (A)** The morphological characteristics of the cell of strain LC-T2^T^ in micrograph. **(B)** Transmission electron micrograph of the cell of strain LC-T2^T^. **(C)** Flagella stain of the cell of strain LC-T2^T^ in micrograph. Cells grown on R2A at 28 [℃](http://www.so.com/link?m=aWv49JHreBMtEOYeGlR%2BH5i6ohE3JCKNdz7InznywGMqqDRndY43SUf5Of7abZIQg5JHSamOwB3j10Ui4O4u0QP87QMJ0uBxhweiltCBjakWIeXxr%2BobWOuBy6id7YF%2BlBnfRNaaPyfTYBc0OheiMSBoB%2Fm%2FRNJJy32G8cgCsdUXJexaDTIl%2Brr0Q49ztW8oTyLdYj5Qps1g%3D) for 3 days. Bar, 0.1 μm.

**Supplementary Figure 4 |** Two dimensional TLC of polar lipids composition of strain LC-T2^T^ after staining with phosphomolybdate reagent **(A)**, molybdenum blue reagent **(B)**, ninhydrin reagent **(C)** and 1-methylnaphthol reagent **(D)**. PE, phosphatidylethanolamine; DPG, diphosphatidylglycerol; PG, phosphatidylglycerol; PL1-3, unidentified phospholipids; APL1-2, unidentified aminophospholipids; GL1, unidentified glycolipid.

**Supplementary Figure 5 |** Effects of LC-T2^T^ bacterization on root activity **(A),** leaf chlorophyⅡ content **(B)** and photosynthesis **(C, D, E, F)** of white clover. Values are means and bars indicate SDs (n = 6). Columns with different letters indicate significant difference among treatments at *P* < 0.05 (ANOVA and Duncan’s post hoc multiple comparison test).

**Supplementary Table 1 |** Draft genome features of *Paenibacillus* *monticola* strain LC-T2^T^

| Attributes (Features) | Values |
| --- | --- |
| Genome size (bp) | 7,082,651 |
| G+C content (%) | 46.0 |
| Total number of genes | 6,236 |
| Protein-coding genes | 5,978 |
| RNAs gene | 112 |
| rRNAs(5S, 16S, 23S) | 23 |
| tRNAs | 85 |
| ncRNA | 4 |
| Pseudo genes | 146 |

**Supplementary Table 2 |** The comparison results of the 16S rRNA gene sequences of isolates that were similar to strain LC-T2^T^ with the same identification results in LC-T2^T^-inoculated rhizosphere soil.

| Hit taxon name | Hit strain name | Accession | Similarity | Diff/Total | Hit taxonomy | Completeness |
| --- | --- | --- | --- | --- | --- | --- |
| *Paenibacillus donghaensis* | KCTC 13049(T) | CP021780 | 97.26 | 39/1423 | Bacteria;Firmicutes;Bacilli;Bacillales;Paenibacillaceae;Paenibacillus | 100 |
| *Paenibacillus odorifer* | DSM 15391(T) | CP009428 | 96.84 | 45/1423 | Bacteria;Firmicutes;Bacilli;Bacillales;Paenibacillaceae;Paenibacillus | 100 |
| [*Paenibacillus wynnii*](https://www.ezbiocloud.net/taxonomy?tn=Paenibacillus%20wynnii) | DSM 18334(T) | JQCR01000002 | 96.56 | 49/1423 | Bacteria;Firmicutes;Bacilli;Bacillales;Paenibacillaceae;Paenibacillus | 100 |
| [*Paenibacillus albidus*](https://www.ezbiocloud.net/taxonomy?tn=Paenibacillus%20albidus) | Q4-3(T) | KY674517 | 96.42 | 51/1423 | Bacteria;Firmicutes;Bacilli;Bacillales;Paenibacillaceae;Paenibacillus | 100 |
| [*Paenibacillus bryophyllum*](https://www.ezbiocloud.net/taxonomy?tn=Paenibacillus%20bryophyllum) | L201(T) | MF040761 | 96.16 | 54/1423 | Bacteria;Firmicutes;Bacilli;Bacillales;Paenibacillaceae;Paenibacillus | 100 |

The comparison results were obtained from EZ_BioCloud_

**Supplementary Table 3 |** The comparison results of the 16S rRNA gene sequences of isolates that were similar to strain GB03 with the same identification results in GB03-inoculated rhizosphere soil.

| Hit taxon name | Hit strain name | Accession | Similarity | Diff/Total | Hit taxonomy | Completeness |
| --- | --- | --- | --- | --- | --- | --- |
| *Bacillus amyloliquefaciens* | DSM 7(T) | FN597644 | 99.94 | 16/1410 | Bacteria;Firmicutes;Bacilli;Bacillales;Bacillaceae;Bacillus | 100 |
| *Bacillus subtilis subsp. subtilis* | NCIB 3610(T) | ABQL01000001 | 99.87 | 15/1410 | Bacteria;Firmicutes;Bacilli;Bacillales;Bacillaceae;Bacillus;Bacillus subtilis | 100 |
| *Bacillus velezensis* | CR-502(T) | AY603658 | 99.41 | 8/1366 | Bacteria;Firmicutes;Bacilli;Bacillales;Bacillaceae;Bacillus | 95.4 |
| *Bacillus subtilis subsp. stercoris* | D7XPN1(T) | JHCA01000027 | 99.28 | 9/1250 | Bacteria;Firmicutes;Bacilli;Bacillales;Bacillaceae;Bacillus;Bacillus subtilis | 86.2 |
| *Bacillus siamensis* | KCTC 13613(T) | AJVF01000043 | 99.15 | 12/1410 | Bacteria;Firmicutes;Bacilli;Bacillales;Bacillaceae;Bacillus | 100 |

The comparison results were obtained from EZ_BioCloud_
